# The Maudsley Biomedical Research Centre (BRC) data linkage service user and carer advisory group: creating and sustaining a successful patient and public involvement group to guide research in a complex area

**DOI:** 10.1186/s40900-019-0152-4

**Published:** 2019-06-11

**Authors:** Amelia Jewell, Megan Pritchard, Katherine Barrett, Patrick Green, Sarah Markham, Sharon McKenzie, Roger Oliver, Maria Wan, Johnny Downs, Robert Stewart

**Affiliations:** 1 South London and Maudsley NHS Foundation Trust, London, UK; 2 Institute of Psychiatry, Psychology and Neuroscience, King’s College London, London, UK

**Keywords:** Patient and public involvement, User involvement, Service user advisory group, Mental health research, Data linkage

## Abstract

**Background:**

Patient and Public Involvement (PPI) in health and social care research has been shown to improve the quality and relevance of research. PPI in data linkage research is important in ensuring the legitimacy of future health informatics initiatives, but remains sparse and under-developed. This article describes the setting up and evaluation of a service user and carer advisory group with the aim of providing feedback and advice to researchers developing or making use of database linkages in the field of mental health.

**Aim:**

The aim of this study is to describe the creation and formative evaluation of the service user and carer advisory group after a trial period of 12 months.

**Method:**

Six individuals were recruited to the group all of whom had personal experience of mental illness. A formative evaluation was conducted after a trial period of 12 months.

**Results:**

Evaluation revealed that the group succeeded in promoting dialogue between service users/carers and researchers. Factors that contributed to the success of the group’s first year included the opportunity it provided for researchers to involve service users and carers in their projects, the training provided to group members, and the openness of researchers to receiving feedback from the group.

**Conclusion:**

The group encourages the incorporation of PPI in data linkage research which helps to ensure the legitimacy of data linkage practices and governance systems whilst also improving the quality and relevance of the research being conducted using linked data.

**Electronic supplementary material:**

The online version of this article (10.1186/s40900-019-0152-4) contains supplementary material, which is available to authorized users.

## Plain English summary

Patient and Public Involvement (PPI) in health and social care research has been shown to improve the quality and relevance of the research being conducted. Data linkage is a technique which is increasingly being used within health research. Data linkage is the joining of information from two or more different sources that relate to the same individual, family, place, or event. PPI is important in research that uses data linkage, but remains sparse and under-developed.

This article describes our experience of setting up a service user and carer advisory group with the aim of providing feedback and advice to researchers developing or making use of database linkages in the field of mental health research. After a trial period of 12 months we conducted an evaluation of the group with both group members and presenters.

We found that the group encouraged researchers to incorporate PPI in their data linkage research and this helps to improve the quality and relevance of the research being conducted using linked data in the area of mental health.

## Background

Patient and Public Involvement (PPI) is defined in the United Kingdom (UK) as research which is carried out ‘with’ or ‘by’ members of the public (including patients and carers) rather than ‘to’, ‘about’, or ‘for’ them [1]. The importance of PPI in health and social care research is widely recognised in the UK [2] and internationally [3]. PPI has been shown to improve the quality and relevance of research and have a positive impact at all stages of the research project [4]. Specifically within health research, service users can bring a personal knowledge of what it is like to live with a specific condition [5]. As observed in Arnstein’s Ladder of Participation [6], there are many degrees of PPI within health and social care services, from non-participation (manipulation or therapy), to tokenism (consultation or placation), and citizen control (partnership or delegated power). Patients and the public can be involved at different stages in the research process which span the full ladder of participation; including identifying research priorities and setting agendas, helping to develop patient information, collecting data, and informing policy [7]. Involving patients and the public in research can be viewed as a way of promoting greater societal awareness and acceptance of research [8] as well as contributing to feelings of empowerment and value for those participating in PII work [9, 10]. In terms of benefits to researchers, patient engagement has been found to aid in securing funding, designing study protocols, and selecting relevant outcomes [11].

Data linkage is the joining of two or more data sets from different sources that relate to the same individual, family, place, or event [12]. Re-using and extending existing routinely collected data through data linkage is a cost effective way of supporting research in public health and epidemiology [13]. It is also an effective way of examining the relationship between health and social factors [14] and data linkage is thus increasingly being used in health research. For instance, health data has previously been linked to a number of non-health data including the UK census [15], education data [16], and prison service data [13].

As well as improving the quality of health research being conducted, patient and public involvement is crucial for ensuring the legitimacy of current practices and governance systems [14]. Furthermore, engaging stakeholders in the development and design of data linkage projects and research questions is an important way of ensuring that the research conducted is relevant to those who are most impacted by it. Despite this, there is currently little public awareness or scrutiny of data linkage, particularly in the area of health research [17]. Data linkage is challenging and, although it is of direct concern to them, members of the public may not be naturally versed in this area. A recent systematic review highlighted the need for greater public awareness around data linkage combined with opportunities for public engagement and deliberation in order to ensure the legitimacy of future health informatics research and for avoiding public controversy [14]. Studies have observed that personal health data are viewed by the public as confidential, private, and sensitive, and should not be shared outside secure, authorised bodies such as the NHS, and especially not with private companies such as employers, insurance providers, and drug manufacturers [17]. Furthermore, differentiations are made between different types of health data with mental health data in particular being viewed as more sensitive [14], therefore warranting involvement from individuals who have particular insight in mental health disorders and related services.

Despite the need and complexity there is very little guidance on how to set up and sustain PPI groups which are well versed in data linkage with only two examples identified in the existing literature [18, 19]. This article describes the setting up and evaluation of a specifically focused service user and carer advisory group with the aim of providing feedback to researchers conducting data linkage projects involving mental healthcare data from the South London and Maudsley NHS Foundation Trust (SLaM) case register. The Maudsley Biomedical Research Centre (BRC) Clinical Record Interactive Search (CRIS) system forms the basis of the SLaM case register and is a de-identified database of electronic medical records from SLaM, described previously in detail [20, 21]. Records from CRIS have previously been linked to a number of other health and non-health databases including the National Pupil Database [16], the Thames Cancer Registry [22], and general practice (GP) data [23]. These linkages are facilitated by the SLaM Clinical Data Linkage Service (CDLS), a trusted third party safe haven set up to facilitate secure data processing services, including data linkage, storage, and extraction [21]. The Maudsley BRC is a partnership between SLaM and the Institute of Psychiatry, Psychology and Neuroscience at King’s College London, funded by the National Institute for Health Research (NIHR). It is one of several national centres set up to bring academic and healthcare institutions together in order to foster translational research [24].

The purpose for setting up a service user and carer advisory group was to ensure that the data linkage research being conducted within the Maudsley BRC is directed by priorities identified by service users with their perspective considered throughout the research project. Therefore, it was agreed that the main aims and objectives of the group would be: (i) to act as a point of contact for researchers seeking service user and carer advice on studies involving data linkage, (ii) to promote the service user/ carer perspective in mental health data linkage studies, (iii) to identify and share good practice in service user and carer involvement, (iv) to build meaningful and reciprocal relationships between researchers and service users when designing studies, preparing grant applications, conducting research, and disseminating findings, and (v) to identify research priorities.

## Method

### Aim

The aim of this study is to describe the creation and formative evaluation of a service user and carer advisory group, set-up for the purpose of providing PPI for mental health data linkage projects being conducted within the Maudsley BRC.

### Setting up the Data Linkage Service User and Carer Advisory Group (DL-SUCAG)

#### Patient and public involvement

Advice was sought in the development of the DL-SUCAG from the Maudsley BRC Service User Advisory Group (SUAG) to ensure the development of the group reflected recognised PPI standards. A proposal to recruit six to eight adult service users/ carers for a pilot period of 12 months was presented to the BRC SUAG in March 2016.

#### Recruitment

The aim of recruitment was to involve a range of individuals, aged 18+, who had experience of mental illness, either themselves or through caring, and who had experience of using NHS mental health services. Members were not expected to be representatives of wider groups. The main source of recruitment to the group was the SLaM Involvement Register, which is open to people who have used SLaM services in the last five years, including their friends, families, and carers. This register is a way for the Trust to advertise and allocate opportunities to people who want to use their experience of using SLaM services to help to improve them in the future [25]. An Involvement Register Activity Criteria was completed, including a brief description of the group, details on the frequency and duration of meetings, training provided, and whether or not any specific skills or abilities were required; this was circulated to members of the SLaM Involvement Register. Group members were offered payment for their time in line with SLaM Involvement Register payment rates as well as reimbursement for travel costs.

#### Initial meetings and training

Although not the case for all, it is generally felt that patients and the public have limited knowledge about data, anonymisation, aggregation, and the regulations surrounding it [26], it was therefore agreed that training should be provided to help contextualise the research being conducted and address concerns that members may have around the use of data. Training sought to provide members with information about the current research context at SLaM, as well as an explanation of technical terms such as ‘data linkage’, information on key research infrastructure (including the CRIS system), and the data protection and information governance procedures in place to protect the personal data of service users. One of the main aims of the training was to ensure that group members understood the governance surrounding data linkage so that they felt confident enough to query researchers on their purpose, designs, and the potential harms of conducting the linkage studies.

Training took place over the first two group meetings (2.5 h in total) and was led by the group facilitators with guest sessions from the Trust Information Governance (IG) Lead and CRIS Academic Lead. All group members attended the training sessions. The first training session took the format of short lectures providing an overview of key research infrastructure and data linkage followed by a group discussion and the opportunity to ask questions. The second session involved a group discussion on information governance and security and the importance of PPI in research. Where possible, real life research examples were used to illustrate concepts and group members were provided with a copy of the training materials for reference (available from the authors upon request).

Terms of Reference for the group were drafted prior to the initial training sessions. These included the aims and objectives of the group as well details on frequency, location, and record keeping. During the initial session the Terms of Reference were reviewed, discussed, and approved by the group.

#### Frequency and composition of meetings

During the pilot year, four meetings were held. Each meeting was 90 min long and consisted of two presentations from researchers conducting data linkage research in the area of mental health. The group was advertised to researchers via word of mouth, posters, and the Maudsley BRC website. Prior to meetings researchers were advised by the group facilitators on the function, aims and objectives, and the level of knowledge of the group to ensure that presentations were appropriate and could be pitched at the right level. Each presentation was followed by a discussion about the project, including an opportunity for group members to ask questions. The discussions enabled the group to give their feedback and opinions on the projects as well as answer any specific questions from researchers. During the meetings formal minutes were taken detailing the researcher presentation and the subsequent discussion that had taken place, these were then circulated to both group members and attendees.

During months without a meeting an email newsletter was sent to all members. The newsletter included information on project or group updates/ news and also highlighted recent publications featuring data linkage research in the area of mental health. It was felt that a newsletter would be a good way of keeping group members up to date with the linkage projects that they had seen, as well as bridging the gap between quarterly meetings.

#### Evaluation of the group

At the end of the pilot year a formative evaluation took place. A discussion group [27] led by the group facilitators was conducted with all six group members with the aim of obtaining feedback on different aspects of the group. The purpose of the discussion was to evaluate the group as well as make decisions to shape the future of the group. Themes for discussion were agreed a priori and included the aims of the group, information provided prior to meetings, format of meetings, researcher presentations, and general feelings towards mental health research using data linkage. The discussion group was recorded and transcribed. A questionnaire was also emailed to all members with specific questions about the format and administration of the meetings (Table 1).

**Table 1:** Survey questions sent to group members

What were your reasons for joining the group?	
Are you happy with the structure of the meeting i.e. short presentation followed by a discussion?	
Do you think the length of the meetings is sufficient?	
Do you think there are a suitable number of members in the group?	
Do you think further training would be useful for members?	
Do you think the presenters described their data linkage projects clearly?	
Any further feedback/ comments about the group in general?	

Researchers who attended the group during the trial year were sent an online survey (Table 2) to complete. A review of the minutes from the meetings which took place during the trial year was also conducted. Feedback from the discussion group and surveys was summarised by the group facilitators in an evaluation report which was circulated to group members for discussion and approval.

**Table 2:** Survey questions sent to presenters

What project did you visit the group about?	
What were your reasons for visiting the group?	
What impact did your visit to the group have on your project?	
Did you find the advice/ feedback provided by the group helpful?	
Would visit the group again in the future?	
Would you recommend the group to other academics conducting data linkage projects?	
Any further comments?	

Findings are reported in line with the Guidance for Reporting Involvement of Patients and the Public (GRIPP2) checklist [28], for the full checklist please see the Additional file 1 Material.

## Results

At the time of the evaluation the group consisted of six working age adults (18+) all of whom had personal experience of mental illness, either as a service user or carer, and who were all either current or past service users of NHS mental health services. All members took part in the discussion group. Following the discussion group a survey was emailed to members (Table 1), only one response to the survey was received.

During the 12 month trial, seven researchers presented at meetings. All presenters were researchers at the Maudsley BRC who were undertaking or planning to undertake projects using mental health data from the CRIS system linked to data from an external source. Five researchers responded to the request for feedback.

### Content of the meetings

The minutes of the meetings were reviewed; during the trial year six data linkage projects were presented to the group. The aims and objectives of the group were agreed with group members at the initial meeting. Throughout the trial year the group were not involved in all of the activities set out in the aims as they were not required by the presenting researchers. Projects presented during the first year included brand new mental health linkages at the start of the approvals process, e.g. a linkage between mental health data and a national audit of heart attacks, and projects being conducted using existing linkages, e.g. a project looking to improve end of life care in dementia using a linkage between mental health and general hospital data. Frequent questions from researchers to the group included “do you think it is acceptable to use patient identifiable information in order to conduct this data linkage?”, “are there any factors which you think I should be focusing on in this analysis?”, and “how can I best communicate these findings to relevant stakeholders?”. Examples of questions, feedback, and opinions from the advisory group to the researchers are presented in Table 3.

**Table 3:** Examples of feedback provided by the group to researchers, taken from the minutes of the meetings

Questions	Will the research look at causes as well as treatment?
	The group asked about the links between self-harm and suicide attempts and how this was being included in the project.
	The group asked whether the study was including sources of patient self-reports?
Feedback	Focusing on medication as treatment alone is too limited. There is a whole social aspect that feeds into the experience of mental health patients.
	The group felt that there were key points in life that would be interesting to focus on. In particular individuals aged between 16 and 24.
	The group raised the issue of non-disclosure that may impact on the records that were being reviewed.
Opinions	Members of the group felt that the aim of the project is of significant public interest.
	Child mental health services were recognised as an important area for studies to take place because of the possibility of intervening early in life to prevent problems later.
	The group thought that as well as informing young people, parents should also be the focus for any communications.

### Members views of the group

The feedback from the discussion group and survey are summarised below.

#### Aims of the group

During the discussion the aims of the group were considered; when asked what they felt the aims of the group were it was agreed that the main aim was to provide a service user perspective to data linkage projects in mental health.

“*To provide a service user input into the data linkage work that you’re doing.*” GM1.

“*To be able to get a different perspective from the service user…*” GM2.

Members felt that it was important and ethical to include a service user perspective as this could provide a very different viewpoint from conventional sources. Members also felt that the group could contribute to issues around confidentiality in research.

*“It’s a focus group, it’s where somebody could actually give their view about what they should be doing perhaps or what areas, because you mentioned confidentiality which is quite an important issue, it could then focus their idea or make it more user friendly in relation to involvement and to get the mind-set that you could involve service users and carers in future with their project without any qualms or preconceptions that it’s going to have barriers or whatever especially with people who haven’t already really been involved with service users previously.”* GM3.

#### Training

The initial introduction and training sessions were discussed. The group felt that these sessions were required for new members as it helped to ensure that everyone was aware of the objectives and purpose of the group.

“*I think it was needed*” GM4.

-*“…it gets everybody in line with what they can give to it because I think if you go in without, (some) people have got their own views and it could be totally alien, it could throw out the group for time because that person may have got their own agenda”* GM3.

It was agreed that the training was sufficient for the current needs of the group; however, it was also agreed that if projects became more complicated then more training may be required. One suggested training area was in research ethics.

“*I think it might be in future if you get some ideas and things it might lead to more training possibly, because people you know are not every day in this research*” GM3.

#### Sharing experiences

Group members recognised that what was being shared during meetings was based on personal experiences and members agreed that they felt comfortable with the confidentiality of what was being said and that it was being kept within the group.

“*I draw on my experience of my own mental ill health*” GM1. -.

“*I’m sure everybody here treats what’s said as confidential and doesn’t take it out of this room and I think it’s an understanding you have*” GM3.

It was noted that sometimes people may worry about oversharing particularly where people might be unhappy about treatment they have received.

*“It’s more that certain subjects are like a red rag to a bull and so when I hear about psychosis your first reaction is to think about your own treatment and what’s happened”* GM1 -.

#### Presentations

It was agreed that the researchers who presented at meetings had been open to the groups feedback and that researchers were happy to give their time. It was also noted that researchers explained their projects clearly to the group and had been happy to explain difficult concepts where needed.

“*I think they’ve very open as well, very open to your views which is really good*” GM2.

“*It’s good because I think they are happy to give time too, like, if you don’t understand they will explain it as well, it’s quite good”* GM5.

During the meetings, members felt that everyone had space to speak. However, some members felt that the meetings could be longer to enable everyone to have their say.

“*I think being here, I think everybody’s had a chance to say their thing and, you know, nobody’s been cut down because we haven’t got time to deal with that, everybody is allowed to have their say and I think that’s part of a good group really*” GM3.

“*Could be longer at times, sometimes as time running out the meetings feel rushed.*” GM1.

Group members also expressed an interest in presenters returning to give updates on their projects.

*“If we could see feedback at other stages even if it’s in narrative form that would be really good because you could see what it’s been used for and how it’s been beneficial to their project that would really be great”* GM3.

#### Thoughts on data linkage

Members felt that as a result of the group they felt positive about the data linkage research being conducted, they also felt able to discuss the group with family and friends.

“*I feel quite positive, especially with the confidentiality of things…*” GM5.

“…*my family tend to know when I go to a meeting and I nearly always have positive things to say about the meeting and I found the meetings engaging and interesting and it’s a good place for discussion”* GM1*.*

### Value of the group to researchers

Feedback from the survey completed by researchers who presented at meetings is summarised below.

#### Reasons for attending

All researchers who completed the survey (Table 2) stated that they attended in order to obtain service user and carer feedback and perspective on their data linkage projects. Other reasons given for attending the group included to gain views on the specific data linkage methodology in their project, understand the privacy concerns, and test the acceptability of linkage methodology.

“*To gain views on the linkage methodology, understand the privacy concerns, how could best engage people on the study process and findings, and whether the questions to be tackled were viewed by the group to be in the patient/public interest.*” R1.

“*To find out how our data linkage can answer research questions of importance to service users and carers, and how it can best inform and improve their services. To discuss the acceptability of this linkage and to take into account and learn from any concerns or comments from service user and carer advisory group.*” R2.

#### Impact of the group

Researchers felt that their visit to the group had a positive impact on their project and all researchers who responded to our survey request felt that the advice and feedback provided by the group was helpful. Survey responses indicated that the feedback received by researchers had had an impact on their projects in a number of different ways, from highlighting particular priority areas to focus on within the analysis to informing information to be included in public information sheets and fair processing notices:

“*Questions asked about the linkage methodology were helpful in framing what to include in information sheets”* R1.

“*Helped prioritise some research question over others.”* R1.

*“Invaluable in supporting my ethics application”* R3.

*“It enabled us to develop our research plan to get the most out of the data linkage for carers and service users.”* R2.

*“It made me realise that I need to talk to carers as part of the project too, to comprehensively understand the social context of mental physical comorbidity”* R4.

One of the projects presented to the group was a data linkage between mental health data and data from a non-health Government department. The researcher leading the project visited the group to discuss the acceptability of the project, specifically the linkage methodology, and to obtain service user and carer input into the specific areas of interest within the project. Subsequently, a Section 251 application was submitted to the Health Research Authority (HRA) Confidentiality Advisory Group (CAG) for the project. Following provisional approval of the Section 251, the HRA CAG provided positive feedback about the DL-SUCAG:

*“The Group commented that the public and patient involvement and engagement which had been undertaken in the project design phase was very strong and provided reassurance for the project. It was acknowledged that the SLaM Trust has an established advisory group, Biomedical Research Centre Data Linkage Service User and Carer Advisory Group. The group comprised of people with lived experience of mental illness that have an interest in mental health research involving data linkage. Members acknowledged that this group had been consulted in the design phase of the project and would continue to be engaged with as the project progressed, which was commended.”*

#### Future visits

All researchers who responded to the survey said that they would visit the group again in the future and everyone who completed the survey said that they would recommend the group to other academics conducting data linkage projects:

*“The group helps motivate me to stay on track and keep pushing projects through to completion. It provides a reality check on what patients’ want to see come from the research we conduct. It helps me revisit the language and explanations I use to describe complex data linkage and governance issues.”* R1.

*“I would definitely, it’s a great group. I think it’s important that the presenter has a very clear question to ask of the group. Otherwise it’s a waste of the resource.”* R5.

“*I think many projects would struggle to get through the approvals stage without the group’s input.*” R3

### Cost of the group

The total cost of the group for the trial year was around £550, this included recruiting to the group, holding the meetings, and remunerating group members. This did not include the time of the members of staff who set up and co-ordinated the group.

## Discussion

This paper describes the setting up and formative evaluation of a service user and carer advisory group (known as the DL-SUCAG) to provide a PPI platform specifically for researchers conducting data linkage projects. As far as we are aware this is the first account on the set-up and evaluation of a regular PPI group in the UK aimed at supporting data linkage research in the area of mental health research. From the perspective of the group’s members, the factors that contributed to the success of the group’s first year included the opportunity it provided for researchers to involve service users and carers in their projects, the level of training provided to the group prior to meetings, and the openness of researchers to receiving suggestions and feedback from the group. With regards to the researchers who attended the group, feedback was positive, with researchers stating that the group helped them to keep in mind the service user perspective within their research and providing a reality check of what patients and the public want to see from mental health research.

Previous research has identified a number of potential barriers to PPI in health research, including the scientific language used in research, lack of support for PPI from research funders, the perception that members of the public have biased views, and the attitudes of researchers to relinquishing power and control [29]. Based on the feedback from group members and researchers, our view is that the group has succeeded in breaking down some of these barriers. For example, during the discussion group it was agreed that researchers had explained their projects clearly and had been happy to explain difficult concepts. The meetings permitted group members to seek clarification from the researchers, to the point where all were satisfied that the project purpose were clear, providing evidence that the group had addressed issues of the use of scientific language in research. Furthermore, it does not cost researchers to attend the group, meaning that support from research funders is not required.

One of the main concerns when setting up the DL-SUCAG was the level of understanding within the group of what might be seen as complex technical processes used within data linkage. Previous research has suggested that the general public feel that the complexities of data linkage research may be difficult to explain in lay terms [19] and that patients and the public have limited knowledge about data, anonymisation, aggregation, and the regulations surrounding these [26]. Therefore, training sessions were set up for all new group members. Training sought to provide members with information about data linkage, including the information governance procedures in place to protect the personal data of service users. The training was discussed at the one-year evaluation and group members concurred that the training sessions were required and important. It was agreed that the training provided was sufficient and this was demonstrated throughout the meetings during the first year where group members were able to be constructively critical of the research projects presented. Our experiences are consistent with a number of previous studies examining public attitudes towards the linkage of health data. A recent systematic review found that studies that followed deliberative qualitative techniques – an approach which allows participants to consider relevant information, discuss issues and develop their thinking [30] – demonstrated that members of the public were competent, able, and enthusiastic to engage in discussions on data sharing and data linkage [14].

Setting up the group and coordinating on-going meetings takes time and effort; however, the financial cost of the group, excluding the time of staff members, was relatively small, at only £550 for the first year. However, the on-going costs of supporting the group may change and will be influenced by its objectives and the criteria for evaluating its usefulness. Despite this, the evaluation revealed a number of less tangible benefits, such as promoting dialogue between service users, carers, and researchers and improving the awareness and perception of data linkage research, which cannot be included in a basic cost analysis. This is consistent with previous literature which has identified a number of benefits of public involvement which cannot be translated into monetary measures including the effects of research design and implementations and the effects on NHS governance and legitimacy [31].

### Impact

Beyond qualitative responses from researchers, the extent to which the group is considered to have had a tangible impact on the research being conducted was difficult to assess. The projects presented to the group were at widely varying stages in the research process, and therefore it was not possible to accurately measure the impact by quantitative means, for example the time to reach ethical approval. However, following the first year evaluation, group members expressed a willingness to continue being involved with the group. There was also a great deal of support from BRC researchers who valued the availability of the group to help with planned and on-going data linkage projects. Group members have developed an expertise in data linkage and mental health research, and there are plans for researchers to return to report back to the group on their projects in order to receive service user and carer input at all stages of their data linkage projects. This is just one possible method of PPI and the appropriateness of different methods for different types of research is something that still needs to be considered [5].

### Limitations

There are a number of limitations to this project. We did not conduct a formal qualitative evaluation of the group and its impact. During discussions with group members it was felt that a formative evaluation would provide the necessary feedback to ensure that the group was meeting its aims and objectives and to evaluate the design of the group in order to make any necessary improvements going forward. Despite this we were able to obtain valuable feedback from both the group members and the researchers who attended. Following the first year evaluation changes to the group were made, including increasing the length of meeting from 90 min to two hours. Going forward, a more in-depth evaluation, including evaluating the group against recognised PPI standards, such as the INVOLVE National Standards for Public Involvement [2], would be useful to ensure the on-going relevance of the group to both the group members and the researchers attending.

Furthermore, we only received one response to the survey which was sent out to group members following the discussion group. However, all group members took part in the discussion group and through this we were able to obtain valuable feedback from group members’ perspectives. Group membership consisted of adults of a range of ages (all 18+) and both genders were represented. Detailed demographic information on group members, e.g. ethnicity, age, etc., was not collected as it was not felt that it was relevant to membership of the group. We therefore cannot comment on the diversity of the group and the group may not be representative of the diverse community of SLaM service users and carers.

Following the trial year the group could be considered as a form of ‘consultation’ according to Arnstein’s ladder of participation, which can be considered a tokenistic form of PPI [6]. However, our hope going forward is that researchers will return regularly to provide updates on their projects and seek the groups input throughout the life of their projects. This is something that both group members and researchers expressed an interest in during the evaluation. We believe in doing so that the relationship between researchers and the group will become a partnership. Although difficult to measure at this stage, going forward, this is something which should be taken into consideration in future evaluations of the group.

## Conclusions

This paper provides a description of how the Data Linkage Service User and Carer Advisory Group was set up operationally within the Maudsley BRC, a joint partnership between a university and healthcare provider. Evaluation of the group after the first year was overwhelmingly positive with the experience being valued by both group members and the researchers who attended the group. Going forward researchers are able to draw on the knowledge of the group throughout the life of their data linkage projects in the area of mental health research. The group helps to maximise the legitimacy of data linkage practices and governance systems by encouraging the inclusion of PPI in data linkage research.

## Additional file

Additional file 1: GRIPP2 Checklist (long form) for Article on DL-SUCAG (DOCX 23 kb)

## Data Availability

Not applicable.

## Abbreviations

BRC: Biomedical Research Centre
CAG: Confidentiality Advisory Group
CDLS: Clinical Data Linkage Service
CRIS: Clinical Records Interactive Search
DL-SUCAG: Data Linkage Service User and Carer Advisory Group
GM: Group Member
GP: General Practice
HRA: Health Research Authority
NHS: National Health Service
NIHR: National Institute for Health Research
PPI: Patient and Public Involvement
R: Researcher
SLaM: The South London and Maudsley NHS Foundation Trust
SUAG: Service User Advisory Group
UK: United Kingdom
